# Bridging cultural gaps in end-of-life care: the experiences of international charge nurses in Saudi Arabia

**DOI:** 10.1186/s12912-024-02514-7

**Published:** 2024-11-28

**Authors:** Zakaria A. MANI

**Affiliations:** 1 Nursing Department, Jazan University, Jazan, Saudi Arabia; 2 School of Nursing and Midwifery, Monash University, Melbourne, Australia

**Keywords:** End-of-life care, Islamic beliefs, Cultural sensitivity, International nurses, Saudi Arabia, Qualitative research

## Abstract

**Introduction:**

This qualitative study explores the experiences and perspectives of international intensive care unit charge nurses providing end-of-life care to Muslim patients in Saudi Arabia. It examines how these nurses navigate the complexities of delivering culturally sensitive care, particularly regarding Islamic beliefs and practices. The study also investigates the challenges encountered by international nurses due to differing healthcare expectations between themselves and patients’ families, highlighting the interplay between cultural sensitivity and effective end-of-life care in this unique context.

**Method:**

A qualitative descriptive design was employed, using semi-structured interviews to gather data from eight international ICU charge nurses working in a tertiary hospital in Saudi Arabia. Thematic analysis was used to analyze the interview transcripts.

**Results:**

This qualitative study explored the experiences of international ICU charge nurses in Saudi Arabia regarding culturally sensitive end-of-life care within Islamic traditions. Analysis revealed nine key themes and 31 subthemes reflecting the multifaceted nature of this sensitive domain. These themes encompassed intercultural anxieties, emotional burdens on families and nurses, the importance of bridging cultural divides, advocating for change in end-of-life care practices, and honoring diverse spiritual needs. Key findings emphasized the significance of family presence, honoring faith in the absence of family, and ensuring peaceful and compassionate passings, highlighting nurses’ commitment to holistic, patient-centered care that respects both cultural and individual beliefs..

**Conclusion:**

This study provides valuable insights into the cultural nuances of end-of-life care in Saudi Arabia. The findings underscore the importance of culturally sensitive practices that respect Islamic beliefs, prioritize family involvement, and address the holistic needs of patients and their families.

**Implications:**

This study underscores the need for culturally sensitive communication training for healthcare providers working with diverse patient populations. Hospitals and healthcare institutions should prioritize educational initiatives that equip staff with the skills to engage in open dialogues about death and dying, navigate cultural differences in end-of-life preferences, and address the use of traditional healing practices. By fostering greater cultural understanding and communication competency, healthcare systems can better support both patients and families in navigating the complexities of end-of-life care.

**Supplementary Information:**

The online version contains supplementary material available at 10.1186/s12912-024-02514-7.

## Introduction

Healthcare providers, particularly nurses, play a crucial role in ensuring the provision of appropriate end-of-life care for patients from diverse cultural backgrounds [1–4]. In the context of the intensive care unit (ICU) in Saudi Arabia, understanding the perceptions and experiences of ICU charge nurses regarding end-of-life care within the Islamic culture is essential for delivering culturally competent care to patients and their families [5–9].

The cultural and religious beliefs of patients and their families can significantly influence their perspectives on end-of-life care decisions [10], particularly in the Saudi Arabian context, where the majority of the population is Muslim [1, 5, 6, 11]. Nurses must be cognizant of these cultural and religious nuances to provide compassionate and sensitive care that aligns with the patient’s and family’s values and preferences [11–16].

International nurses working in Saudi Arabia may experience cultural differences and challenges in navigating the unfamiliar cultural landscape, which can impact their ability to provide culturally appropriate end-of-life care [6, 9, 16–18]. Identifying cultural perspectives in the end of life care and knowing patient and their families needs are necessary to ensure that they receive appropriate and culturally sensitive care during this critical time [1, 19, 20].

By exploring the perceptions and experiences of ICU charge nurses in Saudi Arabia, this research aims to contribute to the understanding of the unique cultural and religious considerations that shape end-of-life care practices within the Islamic context. The findings of this study can inform the development of culturally sensitive strategies and educational programs to enhance the provision of end-of-life care in Saudi Arabian intensive care units. The purpose of this study is to explore international ICU charge nurses’ perceptions of end-of-life care in Islamic culture in Saudi Arabia.

## Methods

This qualitative study employed a descriptive design to explore the experiences and perceptions of international ICU charge nurses regarding end-of-life care for Muslim patients in Saudi Arabia [21, 22]. This methodology aligns with the study’s aim to gain an in-depth understanding of the nurses’ perspectives and experiences in this specific cultural context. This study adhered to the Consolidated Criteria for Reporting Qualitative Research guidelines [23]. Specific details regarding each COREQ item can be found in the supplementary materials.

### Interview questions

An interview guide was developed based on a comprehensive review of relevant literature and addressed key aspects of end-of-life care for Muslim patients in the ICU including the findings of Mani & Ibrahim (2017) [7]. The guide included open-ended questions and prompts designed to elicit detailed descriptions of participants’ experiences, beliefs, and perceptions. Key topics explored during the interviews included:

- **Understanding Islamic Perspectives**: Participants were asked about their understanding of Islamic beliefs and practices related to death and dying, including common rituals, family involvement, and spiritual considerations.
- **Experiences with End-of-Life Care**: Nurses were invited to share their experiences providing end-of-life care to Muslim patients, including specific situations, challenges encountered, and memorable interactions.
- **Cultural Sensitivity in Practice**: Participants were asked to describe how they incorporated cultural sensitivity into their care practices, including communication strategies, family interactions, and addressing spiritual needs.
- **Ethical Considerations**: The interview guide explored ethical considerations related to end-of-life decision-making for Muslim patients, such as navigating cultural differences in preferences for life-sustaining treatments or disclosing prognosis.
- **Recommendations for Improvement**: Participants were encouraged to share their insights and recommendations for improving end-of-life care practices for Muslim patients in the ICU.

### Inclusion criteria

This study included international key informants from different countries, specifically managers or charge nurses, as they were assumed to have greater responsibility for and experience with end-of-life care in the ICU in Saudi Arabia. Those eligible key informants who agreed to participate will be included in the study.

### Sampling and sample size

A purposive sampling of key informants was employed to ensure the inclusion of international ICU charge nurses from different countries with significant experience and nuanced understanding of end-of-life care practices within the Saudi Arabian cultural context. This approach was deemed most appropriate for gaining in-depth insights into the specific research questions.

The data saturation was achieved after interviewing seven participants. However, to ensure thoroughness and capture any potential remaining nuances within the data, one additional interview was conducted, resulting in a final sample size of eight. This approach, common in qualitative research, helps to confirm that saturation has indeed been reached and that the data encompasses a comprehensive range of perspectives on the phenomenon under investigation [24]. This saturation point, reached with a relatively small sample size, highlights the homogeneity of experiences among this specific group of nurses working within a particular cultural context and specialized area of care [24].

### Data collection

In-depth, semi-structured interviews were conducted with each participant to explore their experiences and perspectives. This approach allowed for flexibility in probing emerging themes and gaining a deeper understanding of the nuances within their narratives. Interviews were conducted in a private setting at the participants’ convenience, typically during their off-duty hours at the hospital or a mutually agreed upon location. This ensured participant comfort and minimized disruptions to their work schedule.

The interviews for this study were conducted by the main author who holds a PhD in Nursing with expertise in critical care areas. This background provided a strong foundation for understanding and interpreting the gaps of cultural sensitivity in healthcare settings. Prior to the interview, participants received a written information sheet outlining the study’s purpose, procedures, and their rights, including the voluntary nature of participation and the option to withdraw at any time. Informed consent was obtained from each participant before commencing the interview.

All interviews were audio-recorded using digital recording devices to ensure accuracy and allow for verbatim transcription. The average interview duration was 17–35 min. Field notes were taken during and after each interview to capture contextual information, non-verbal cues, and initial impressions. These notes supplemented the audio recordings and provided a richer understanding of the interview context. While all participants were fluent in English, it was not their native language.

To ensure participant confidentiality, all identifying information was removed from the transcripts and replaced with unique identifiers. Audio recordings and transcripts were stored securely in password-protected files, accessible only to the research team.

### Data analysis

Data analysis was an iterative and comprehensive process, guided by the principles of thematic analysis as outlined by Braun and Clarke in the following six steps [25, 26]. This rigorous approach allowed for a rich exploration of the data, moving beyond simple description to identify, analyze, and report patterns within the participants’ experiences and perspectives [25].

1. **Familiarization with the Data**: The initial step involved immersing author self in the data. This began by listening to the audio recordings multiple times to gain a thorough understanding of the content and context of each interview. Then the interviews verbatim were transcribed, ensuring accuracy and capturing nuances in language and tone. Reading and re-reading the transcripts allowed to become intimately familiar with the participants’ narratives and identify initial impressions.
2. **Generating Initial Codes**: This study employed an inductive coding approach, allowing codes to emerge organically from the data rather than imposing pre-determined categories. Two researchers independently read through the transcripts line-by-line, identifying and highlighting segments of text that captured key ideas, concepts, or experiences related to end-of-life care for Muslim patients. These segments were then assigned concise, descriptive codes that captured the essence of the meaning conveyed.
3. **Searching for Themes**: Once the initial coding was complete, it begans with the process of grouping and organizing the codes into potential themes. This involved comparing and contrasting codes across different interviews, identifying patterns and connections, and looking for recurring ideas or experiences that reflected a central organizing concept. NVivo software (version 13) was used to facilitate data management and organization during this stage, allowing to easily group and categorize codes, identify relationships between them, and visually map emerging themes.
4. **Reviewing Themes**: The emerging themes were then critically reviewed and refined through an iterative process of discussion and consensus among the research team. The coherence of each theme was examined, ensuring that the codes grouped within it formed a cohesive and meaningful category. Themes were revised, split, or merged as needed to ensure clarity and accuracy in representing the data.
5. **Defining and Naming Themes**: Once the themes were finalized, clear and concise definitions for each theme were developed, capturing its core meaning and scope. Evocative and descriptive names for the themes that accurately reflected the essence of the participants’ experiences and perspectives were carefully selected.
6. **Producing the Report**: The final stage involved weaving the themes into a comprehensive and engaging narrative that captured the richness and complexity of the data. Illustrative quotes from the participants’ narratives used to provide context and support for each theme, allowing their voices and experiences to shine through. The report aimed to provide a nuanced and insightful account of the experiences of international ICU charge nurses providing end-of-life care to Muslim patients in Saudi Arabia.

### Trustworthiness

Trustworthiness and rigor were central to the qualitative approach. Several strategies were employed to ensure the credibility, transferability, dependability, and confirmability of the findings. Prolonged engagement with data and in-depth interviews, combined with data triangulation using transcripts and field notes, provided a rich understanding of their experiences [21, 22].

A pilot test of the interview questions was conducted to ensure they were culturally appropriate and elicited the necessary depth of information for the study. This involved reviewing the questions with cultural experts and conducting preliminary interviews with a small group of nurses similar to the study participants. The feedback from the pilot test was used to refine the questions and ensure their clarity, relevance, and cultural sensitivity.

Allowing participants to verify the interpretations was crucial to enhance the credibility and confirmability. Thick descriptions of the participants, context, and findings provide transparency and enable readers to assess the transferability of the findings to other settings.

To ensure dependability, a detailed audit trail throughout the research process was maintained, documenting all decisions and procedures. These rigorous practices, grounded in established qualitative research principles, strengthen the trustworthiness and credibility of the findings, offering valuable insights into the experiences of international ICU charge nurses providing end-of-life care to Muslim patients in Saudi Arabia.

### Findings

The study included eight charge nurses, six were female and two were male representing a diverse range of ICU specialties. All participants held a bachelor’s degree in nursing and had significant experience in the ICU, ranging from 5 to 15 years and represented a mix of nationalities (see Table 1).

**Table 1:** Participant profiles

Name (pseudonym)	Gender	Age group	Experience (years)	Qualification	Department	Nationality
Participant 1	F	26–30	5	BSN	Chronic ICU	Malaysia
Participant 2	F	31–35	9	BSN	Surgical ICU	Philippines
Participant 3	F	36–40	15	BSN	Neuro	India
Participant 4	F	31–35	10	BSN	Medical ICU	Philippines
Participant 5	M	36–40	12	BSN	Surgical ICU	Philippines
Participant 6	F	31–35	9	BSN	Medical ICU	Malaysia
Participant 7	F	26–30	8	BSN	Medical ICU	India
Participant 8	M	36–40	13	BSN	Medical ICU	India

End-of-life care within intensive care units presents unique challenges, particularly when navigating diverse cultural and religious landscapes. This study delves into the experiences of international ICU charge nurses in Saudi Arabia, exploring their perspectives on providing culturally sensitive end-of-life care within the context of Islamic traditions. Through qualitative analysis, nine key themes emerged, encompassing 31 subthemes, which shed light on the multifaceted dimensions of this sensitive domain (see Table 2). These themes range from the unspoken anxieties and clashing worldviews that can arise in intercultural care settings, to the profound emotional burdens faced by both families and nurses. The study also highlights the importance of bridging cultural divides, advocating for change in end-of-life care practices, and honoring the diverse spiritual needs of patients and their families. The themes of family presence, honoring faith in the absence of family, and ensuring peaceful and compassionate passings underscore the nurses’ commitment to providing holistic, patient-centered care that respects both cultural and individual beliefs. This research offers valuable insights into the complexities of end-of-life care in a multicultural setting and underscores the need for ongoing dialogue and reflection to enhance the quality and sensitivity of care provided to patients and their families during this critical life transition.

**Table 2:** Emerged themes and sub themes

Themes	Sub themes
The Weight of Unspoken Truths	• Cultural Barriers to Direct Communication • Doctor Avoidance and Family Distress. • Variability in Physician Approaches • Need for Culturally Sensitive Communication Training
Clashing Worldviews	• Traditional Healing Practices vs. Modern Medicine • Respecting Beliefs vs. Ensuring Patient Safety • Practical Challenges for Nurses • Need for Open Communication and Education
The Family’s Agony	• Unintentional Prolongation of Suffering • Seeking Alternative Treatments • Family Discord and Conflicting Requests • Need for Compassionate Communication and Support
The Burden on Nurses	• Emotional Toll of End-of-Life Care • Lack of Emotional Support • Risk of Burnout. • Lack of Clear Protocols • Dedication Despite Challenges
Bridging the Divide	• Patient-Centered Care and Shared Decision-Making • Cultural Sensitivity and Communication
A Call for Change	• Need for Culturally Sensitive Protocols and Training
The Importance of Family Presence	• Shifting from Clinical to Personal • Comfort and Security through Family • Respect for Emotional and Spiritual Well-being
Honoring Faith in Absence	• Providing Spiritual Comfort • Facilitating Family Connections • Respect for Cultural and Spiritual Beliefs
Peaceful and Compassionate Passings	• Holistic Understanding of a Good Death • Compassionate Accompaniment • Pain Management as a Cornerstone of Care • Respect for Patient Autonomy • Dignity as a Guiding Principle

### The weight of unspoken truths

The following quotes highlight the complex interplay between cultural sensitivities, communication barriers, and end-of-life care, particularly the challenging situation where doctors feel compelled to avoid direct conversations about death.

The first two quotes, *“Doctors often avoid telling families their loved one is dying because of cultural sensitivities”* [Participant 3] and *“The doctor avoidance along with the family’s strong faith*,* makes things very challenging”* [Participant 4], reveal a common, yet problematic, dynamic. Doctors, aware of cultural beliefs where directness about death is deemed inappropriate or even harmful, may resort to indirect communication or avoid the topic altogether. While motivated by a desire to respect cultural values, this avoidance can inadvertently create more challenges. Families, left in the dark about the true prognosis, may struggle to make informed decisions or find themselves unprepared for the eventuality of death. This situation can also lead to distrust between medical professionals and families, hindering the collaborative approach essential for optimal end-of-life care.

The third quote, “Few doctors avoid end-of-life care discussion” [Participant 8], presents a contrasting perspective, suggesting that not all doctors shy away from these difficult conversations. This difference in approach could stem from various factors, including individual communication styles, personal beliefs, or experiences with diverse patient populations. It also highlights the need for greater consistency and clarity in how healthcare providers navigate cultural sensitivities while ensuring open and honest communication with patients and families facing end-of-life situations.

These contrasting viewpoints underscore the need for culturally sensitive communication training for medical professionals. Such training should equip them with the skills to engage in open and honest dialogues about death and dying, even within cultures where these topics are considered taboo. Finding a balance between respecting cultural beliefs and providing clear, compassionate information is crucial for ensuring that patients and families feel supported and empowered to make informed decisions about end-of-life care.

### Clashing worldviews

The following quotes reveal the tensions that arise when traditional healing practices intersect with modern medical approaches, particularly in the context of end-of-life care. While cultural sensitivity is paramount, healthcare providers often find themselves navigating a delicate balance between respecting deeply held beliefs and advocating for evidence-based practices.

The first quote, “Families sometimes use honey and oil on infected wounds, believing it will help” [Participant 1], highlights the prevalence of traditional remedies, often passed down through generations, in managing illness and injury. These practices often stem from a deep-rooted cultural understanding of health and healing, holding significant meaning and importance for families.

However, as the second quote, “We tell family that using honey and oil are harmful, but doctors often allow it out of respect for their beliefs” [Participant 4], reveals, such practices can clash with modern medical understanding. While honey possesses some known antibacterial properties, its application to infected wounds might be contraindicated or even harmful in certain medical contexts. This discrepancy creates a dilemma for healthcare providers, caught between advocating for evidence-based care and respecting cultural beliefs that might contradict those recommendations. The decision to “allow” such practices, while seemingly respectful, can be fraught with ethical implications, potentially compromising patient safety and well-being.

The third quote, “Using stuff from their beliefs like oil and water makes our job as nurses harder” [Participant 6], underscores the practical challenges faced by healthcare providers in such situations. Nurses, often tasked with the hands-on care of patients, may find themselves torn between following medical protocols and accommodating family requests that they perceive as potentially harmful. This tension can lead to moral distress, impacting job satisfaction and potentially compromising the quality of care provided.

These quotes highlight the need for open and honest communication between healthcare providers, patients, and families about the efficacy and potential risks of traditional healing practices. Rather than dismissing these practices outright, a more constructive approach involves engaging in respectful dialogue, acknowledging the cultural significance of such beliefs, and explaining the medical rationale behind alternative treatments. Finding a balance between cultural sensitivity and evidence-based practice is crucial for building trust and ensuring optimal care for patients from diverse cultural backgrounds.

### The family’s agony

The following quotes offer a raw and honest look at the complexities surrounding family dynamics in end-of-life care. While motivated by love and a desire to help, families can sometimes inadvertently contribute to a patient’s suffering, highlighting the delicate balance between honoring their wishes and advocating for the patient’s best interests.

The statement, “Families often interfere with care, unaware of their loved one’s true suffering” [Participant 7] reveals a heartbreaking disconnect. Driven by love and a refusal to accept the inevitable, families may advocate for continued interventions, unaware that these actions might be prolonging their loved one’s suffering rather than alleviating it. This disconnect underscores the importance of clear and compassionate communication between nurses and families, ensuring everyone understands the realities of the patient’s condition and the potential consequences of various treatment options.

The quotes, “Families asking different questions about using other treatments plans” [Participant 2] and “Families suggesting different treatments from internet” [Participant 3] further illustrate the emotional turmoil families experience. Desperate for a solution, families may turn to alternative treatments or cling to unrealistic hopes fueled by information gleaned from the internet. While understandable, these actions can create tension and complicate the care plan, potentially leading to disagreements between family members and healthcare providers.

The statement, “Each family member might request a different care plan, prolonging the patient’s suffering” [Participant 7] “sometimes familes members ask for different treartment” [Participant 4] highlights the potential for discord within families grappling with loss. Grief manifests differently in everyone, and family members may disagree on the best course of action for their loved one. These disagreements, while rooted in love and concern, can create a chaotic and emotionally charged environment that ultimately prolongs the patient’s suffering.

These quotes underscore the need for healthcare providers to approach end-of-life care with sensitivity, compassion, and a deep understanding of family dynamics. Open and honest communication, active listening, and empathy are crucial for navigating these challenging situations. Providing families with emotional support, clear explanations, and opportunities to express their concerns can help bridge the gap in understanding and facilitate shared decision-making that prioritizes the patient’s well-being and honors their wishes.

### The burden on nurses

These quotes offer a poignant glimpse into the emotional burden shouldered by nurses, particularly in the context of end-of-life care. The nurses’ words reveal a profession grappling with the profound impact of witnessing suffering and death, often without adequate support systems in place.

The phrase “Nurses bear a heavy emotional burden” [Participant 1] speaks to the inherent weight of accompanying patients through their final moments. This burden is not merely a matter of professional duty but a deeply personal experience that can leave nurses emotionally drained and vulnerable. The acknowledgment that “this burden can impact the quality of end-of-life care” [Participant 3] further underscores the potential consequences for both patients and providers. When nurses are stretched thin emotionally, their capacity for empathy and compassionate care can be compromised, potentially affecting the quality of care delivered.

The pleas for emotional support, “We need emotional support” [Participant 6] and “Sometimes we feel that we need help ourselves” [Participant 8] highlight a critical gap in the system. These nurses are expressing a need for resources and support mechanisms that acknowledge and address the emotional toll of their work. The absence of such support leaves them feeling overwhelmed and ill-equipped to process the emotional intensity of end-of-life care.

The statement “Nurses are at risk of burning out” [Participant 2] serves as a stark warning. Burnout, a state of emotional, physical, and mental exhaustion caused by prolonged exposure to overwhelming stressors, is a serious concern in healthcare professions. The nurses’ words suggest that without adequate support and resources, they are at increased risk of experiencing burnout, which can have detrimental effects on their well-being and their ability to provide quality care.

The lack of clear protocols, as expressed in “We lack specific protocols for end-of-life care in ICU” [Participant 4], “End of life care protocol that employed different cultural and beliefs aspects for new staff are needed” [Participant 5] further exacerbates this burden. Clear protocols can provide a sense of structure and guidance, particularly in emotionally charged situations. The absence of such protocols can leave nurses feeling uncertain and ill-prepared, adding to their stress and anxiety.

The statement “We strive to provide the best support possible” [Particpant 7] reflects the nurses’ dedication to their patients despite these challenges. They are striving to provide compassionate care even in the face of emotional exhaustion and systemic gaps. However, this striving, without adequate support, is unsustainable in the long run.

These quotes, taken together, paint a concerning picture of a system that needs improvement to adequately support its healthcare providers. Addressing the emotional needs of nurses is not merely a matter of improving working conditions; it is essential for ensuring the delivery of high-quality, compassionate end-of-life care. Investing in emotional support resources, developing clear protocols, and fostering a culture of support within healthcare settings are crucial steps towards mitigating the emotional burden on nurses and ensuring their well-being.

### Bridging the divide

These quotes offer a heartening counterpoint to the challenges discussed previously, highlighting proactive steps towards more compassionate and culturally sensitive end-of-life care. They emphasize the power of knowledge, communication, and cultural understanding in navigating this sensitive terrain.

The statement, “Educating patients and families about their options and involving them in the decision-making process is crucial” [Participant 6] and “Given more attention for families are supportive” [Participant 1] underscore a critical shift towards patient-centered care. By empowering families with knowledge about the patient’s condition, prognosis, and available options, healthcare providers can foster a sense of agency and shared decision-making. This approach not only respects patient autonomy but also helps to alleviate the fear and uncertainty that often accompany end-of-life situations.

Recognizing the potential for communication barriers, as expressed in “While we don’t all speak Arabic, translators can bridge the communication gap” [Participant 6] and “sometimes we need translator and also ensuring some sensitive cultural aspects” [Participant 8] demonstrate a commitment to cultural sensitivity. Language barriers can significantly hinder understanding and create a sense of isolation for patients and families. By providing access to culturally competent interpreters, healthcare providers can ensure that critical information is conveyed accurately and respectfully, fostering trust and facilitating meaningful dialogue.

These quotes, while brief, offer a powerful message of hope. They suggest that by embracing open communication, prioritizing patient education, and valuing cultural understanding, healthcare providers can create a more compassionate and supportive environment for patients and families facing the end of life. These efforts, while not always easy, are essential for ensuring that patients receive care that honors their wishes, respects their cultural beliefs, and prioritizes their comfort and dignity.

### A call for change

Ultimately, these quotes “Cultural congruent in end of life is very essintial” [Participant 4], “Considering patients and their families belifs at end of life need attention” [Participant 3], “The culure is very sensitive and very imprtant at the end of life” [Participant 2] and “Most of families have strong faith and beliefs” serve as a powerful call for systemic change. Healthcare institutions must develop clear, culturally sensitive protocols for end-of-life care that address the unique needs of diverse patient populations. This includes providing adequate training for healthcare providers on cultural competency, effective communication strategies, and managing their own emotional well-being. By fostering a culture of empathy, respect, and open communication, we can create a healthcare system that honors both the medical and spiritual needs of patients and their families during this vulnerable time.

### The importance of family presence

These quotes beautifully illustrate the deep understanding these nurses have of the human experience at the end of life. They recognize that death is not just a biological event but a deeply personal and spiritual journey, best navigated with the love and support of close relationships.

The statement, “We generally encourage families to be present with their loved ones” [Participant 2] and “Facilitating family presence” [Participant 4] reflect a move away from a purely clinical approach to end-of-life care. It acknowledges the profound emotional and spiritual needs of patients facing their mortality and prioritizes creating a space where those needs can be met through the comfort of family presence.

The quotes, “Patients mostly trust their families” [Participant 5] and “family present are very supportive for patients” [Participant 7] further highlight the vital role families play in providing comfort and security during a vulnerable time. This trust and support can be immensely powerful, offering patients a sense of peace and connection that transcends the medical aspects of their care. By actively encouraging and facilitating family presence, these nurses demonstrate a deep respect for the patient’s emotional and spiritual well-being. They recognize that the final moments of life are not just about medical interventions but about love, connection, and shared humanity. This approach prioritizes creating a compassionate and supportive environment where patients can find solace and meaning in the presence of their loved ones.

### Honouring faith in absence

The following quotes reveal a commendable dedication to providing holistic care, recognizing that spiritual well-being is just as important as physical comfort, especially at the end of life. Even when families cannot be present, these nurses demonstrate a proactive approach to ensuring their patients feel a sense of peace and spiritual support.

The quote, “If the family is absent, we’ll play Quran TV” [Participant 2] and “Providing Quran to patients and their families” [Participant 8] exemplify a simple yet powerful gesture of respect for the patient’s faith. By providing access to religious content, the nurses create a comforting and familiar atmosphere for the patient, even in the absence of loved ones.

The efforts to “contact the family and encourage them to come” [Participant 1] further highlight the nurses’ commitment to family involvement whenever possible. They recognize the importance of family connections, especially during such a critical time, and actively work to facilitate those connections.

The practice of “Having a religious leader to pray with the patient” [Participant 4] and “Religious leaders are very supportive” [Participant 3] demonstrate a deep understanding of the patient’s spiritual needs. By involving religious leaders, the nurses provide an additional layer of support and comfort, allowing the patient to connect with their faith and find solace in prayer and spiritual guidance.

These actions, taken together, paint a picture of compassionate care that extends beyond the purely medical. The nurses’ commitment to honoring the patient’s faith, even in the absence of family, reflects a deep respect for their cultural and spiritual beliefs, ensuring that they feel seen, heard, and supported in their final moments.

### Peaceful and compassionate passings

The statements, “We advocate for peaceful deaths” [Participant 6], “Free from suffering for both patients and their families” [Participant 2] and “Ideally, death should be comfortable and pain-free” [Participant 6]. articulate a profound shift in the approach to end-of-life care. This perspective transcends the traditional medical focus on cure and life extension, embracing instead a more holistic understanding of a good death. It acknowledges that dying is not just a biological event but a deeply personal and relational process, one that demands compassionate accompaniment for both the patient and their loved ones. The emphasis on minimizing suffering, both physical and emotional, speaks to a deep empathy for the multifaceted challenges faced by those nearing the end of life.

The quotes, “Our focus is on managing pain” [Participant 8], “Meeting patients wishes” [Participant 1] and “Attending and meeting patients’ needs” [Participant 4] translate this compassionate philosophy into concrete actions. Pain management is elevated from a mere clinical task to a cornerstone of care, reflecting an understanding that unrelieved pain can deeply impact not just physical comfort but also emotional and spiritual well-being. The emphasis on “meeting patients wishes” [Participant 3] speaks to a deep respect for patient autonomy, recognizing that individuals have the right to make choices about their own dying process, even when those choices might differ from medical recommendations. Finally, the commitment to “Attending and meeting patients needs” [Participant 6] underscores a holistic understanding of care that extends beyond treating physical symptoms to addressing the emotional, spiritual, and social dimensions of the human experience.

This patient-centred approach finds further expression in the statements, “Maintaining the dignity and respect of patients and their families is paramount” [Participant 2] and “Our goal is to provide comfort, dignity, and a peaceful passing” [Participant 7]. Here, dignity emerges as a guiding principle, shaping every aspect of care. It’s a recognition that each human life, regardless of its proximity to death, possesses inherent worth and deserves to be treated with respect and compassion. This commitment to preserving dignity extends beyond the physical aspects of care to encompass emotional and spiritual well-being, ensuring that patients feel valued, heard, and supported throughout their final journey.

## Discussion

This qualitative study explored the perceptions of international ICU charge nurses regarding end-of-life care within the context of Islamic culture in Saudi Arabia. The findings offer valuable insights into the complexities of providing culturally sensitive care at the intersection of diverse beliefs and practices. While the nurses demonstrated a deep commitment to compassionate, holistic care, their narratives also revealed challenges in navigating cultural differences and balancing medical practices with religious beliefs.

A prominent theme emerging from the data is the paramount importance of family presence during a loved one’s final moments. The nurses consistently emphasized encouraging and facilitating family involvement, recognizing the profound significance of shared experience during this critical life transition. This finding aligns with existing literature highlighting the centrality of family in Islamic culture and the importance of family support in end-of-life care within collectivist cultures [8, 9, 16, 27]. Unlike Western contexts, where individual autonomy often takes precedence [13, 28], Islamic medical ethics emphasizes shared decision-making, with family playing a central role in medical decisions, particularly at the end of life [9, 16, 29].

However, the nurses also acknowledged situations where family presence is not feasible, highlighting their proactive approach to providing spiritual comfort through means such as Quran recitations or involving religious leaders. This proactive approach reflects a commendable sensitivity to the Islamic faith and a commitment to honoring patients’ spiritual needs, even in the absence of family. This finding resonates with literature emphasizing the importance of religious coping mechanisms for Muslim patients facing serious illness [9, 27]. By taking the initiative to provide spiritual support, the nurses demonstrated an understanding that culturally sensitive care extends beyond physical comfort to encompass spiritual well-being, particularly within a religiously diverse ICU environment [1, 20, 30].

Furthermore, the nurses emphasized a multifaceted approach to care, prioritizing pain management alongside family presence and emotional support. This holistic perspective aligns with the Islamic emphasis on alleviating suffering and promoting a peaceful death, as reflected in religious texts and teachings [11, 29]. The nurses’ commitment to advocating for “peaceful deaths, free from suffering” for both patients and families underscores the importance of addressing not only physical pain but also emotional and spiritual distress. This finding resonates with research highlighting the importance of a palliative approach to end-of-life care that prioritizes comfort and dignity for both patients and their families [4, 20, 31].

Despite their dedication to providing culturally sensitive care, the nurses’ narratives also hinted at the complexities of navigating cultural differences and potential ethical dilemmas. Future research could delve deeper into these challenges, exploring how international nurses navigate situations where cultural beliefs might conflict with established medical practices. For instance, further investigation into how nurses balance respecting traditional healing practices with advocating for evidence-based medicine would be valuable. Additionally, exploring the perspectives of Saudi healthcare providers, patients, and families would provide a more comprehensive understanding of end-of-life care within this specific cultural context.

The nurses’ emphasis on incorporating religious practices, such as prayer and Quran recitation, into end-of-life care demonstrates commendable cultural sensitivity. Evidence about culturally sensitive care at the end of life in specific context can enhance nurses understanding and improve the quality of care. Additionally, exploring the potential diversity of beliefs and practices within Islam cultural contexts as discussed in the literature [8, 9, 11, 13, 27], would provide a more comprehensive understanding of patient and family needs. However, future research could benefit from engaging with religious scholars and experts to ensure a nuanced understanding and representation of Islamic perspectives on end-of-life care.

The emotional burden described by the nurses in this study, characterized by the heavy emotional burden and its potential impact on the quality of end-of-life care, aligns with existing literature on the emotional toll of nursing, particularly in intensive care settings. Studies have consistently shown that nurses working with dying patients experience high levels of stress, compassion fatigue, and burnout [32–34]. This emotional strain can negatively affect not only the nurses’ well-being but also the quality of care provided, as emotional exhaustion can diminish empathy and the ability to provide compassionate support to patients and families. The findings underscore the need for greater attention to the emotional support and well-being of nurses working in end-of-life care, including access to resources and strategies for managing emotional stress and promoting resilience. This could involve implementing support programs, promoting a culture of open communication about emotional challenges, and ensuring adequate staffing levels to reduce workload and stress.

Several strategies can mitigate cultural differences in the ICU during end-of-life care. Providing access to trained interpreters or language services can facilitate communication and ensure that patients and families understand medical information and treatment options. Hospitals can also establish culturally sensitive policies and procedures for end-of-life care, taking into account diverse religious and spiritual beliefs. Furthermore, institutional support for cultural competency training for healthcare providers can equip staff with the skills and knowledge to navigate cultural differences effectively and provide respectful, patient-centered care [34].

The nurses’ call for culturally congruent end-of-life care resonates with existing literature emphasizing the need for healthcare systems to adapt to the diverse cultural and spiritual needs of patients. The quotes highlighting the essential nature of cultural sensitivity, the importance of respecting patient and family beliefs, and the acknowledgment of strong faith traditions underscore a need for systemic change within healthcare institutions. This aligns with research advocating for the development of culturally sensitive protocols, training in cultural competency for healthcare providers, and the creation of a healthcare environment that supports both the medical and spiritual dimensions of end-of-life care [33, 34]. Such changes are crucial for ensuring that care practices are respectful, responsive, and truly patient-centered.

This study highlighted the ethical tension between respecting cultural practices and ensuring medical safety, a complex issue frequently encountered in end-of-life care. Navigating this tension requires careful consideration of patient autonomy, cultural sensitivity, and evidence-based medical practice. Open communication, shared decision-making, and a willingness to understand diverse perspectives are crucial for finding a balance that honors both cultural values and patient well-being.

Future research should explore the voices of Muslim patients, families, and other healthcare providers involved in end-of-life care would provide valuable insights into their experiences and needs. Developing and evaluating culturally tailored interventions, such as educational materials or communication tools, could enhance the quality of end-of-life care for Muslim patients. Further research is also needed to investigate ethical dilemmas encountered by healthcare providers and explore strategies for navigating cultural differences in end-of-life decision-making. Finally, conducting longitudinal studies to examine the long-term impact of culturally sensitive end-of-life care on patient and family outcomes would contribute valuable evidence to inform practice.

### Implications

This study’s findings carry significant implications for healthcare practice, education, and policy, particularly in settings characterized by cultural diversity. The narratives of these international ICU charge nurses highlight a crucial need for bridging the gap between respecting cultural beliefs and ensuring clear, honest communication about end-of-life care.

#### Culturally sensitive communication training

A recurring challenge highlighted by the nurses involves navigating cultural sensitivities surrounding death and dying. While motivated by respect for cultural values, avoiding direct conversations about prognosis can inadvertently create communication barriers and hinder informed decision-making. Hospitals and healthcare institutions should prioritize communication skills training for all staff, equipping them with the tools to engage in open and honest dialogues about end-of-life care, even when cultural norms discourage directness. This training should move beyond general cultural sensitivity to incorporate practical strategies for discussing sensitive topics like prognosis, goals of care, and the use of life-sustaining treatments in a manner that respects individual beliefs and values.

#### Integrating traditional healing practices

The nurses’ experiences also highlight the complexities of navigating the intersection of traditional healing practices and modern medical approaches. Dismissing these practices outright risks alienating families and undermining trust. Instead, healthcare providers should adopt an approach of open dialogue and cultural humility. This involves initiating respectful conversations with patients and families about their use of traditional remedies, seeking to understand the meaning and significance behind these practices. Providing evidence-based information about potential benefits and risks of both traditional and modern approaches is crucial, framing this information within the context of cultural beliefs. Where appropriate, exploring ways to safely and respectfully integrate traditional healing practices into the patient’s overall care plan can foster a sense of collaboration and trust.

#### Policy and system-level changes

Creating a culturally sensitive and responsive healthcare system requires changes that extend beyond individual provider behavior. Institutions should prioritize the recruitment and retention of a diverse healthcare workforce that reflects the cultural makeup of the patient population. This diversity, encompassing language, cultural background, and familiarity with traditional healing practices, can help bridge cultural gaps and enhance communication. Ensuring access to qualified interpreters for patients with limited English proficiency is paramount, facilitating clear communication and informed decision-making. Furthermore, integrating cultural sensitivity training into the curriculum of medical, nursing, and allied health professional schools is essential, fostering a workforce equipped to provide culturally competent care from the outset of their careers.

By implementing these recommendations, healthcare systems can move towards a more equitable and patient-centered approach to end-of-life care, one that respects cultural values while upholding the highest standards of medical practice.

## Conclusion

This study offers valuable insights into end-of-life care in Saudi Arabian ICUs from the perspective of international charge nurses. The findings highlight the need for culturally sensitive practices respecting Islamic beliefs and family involvement. Future research should explore these themes with a more diverse sample, including local Saudi nurses, to broaden understanding and address the current study’s limitations.

## Limitations

This study provides valuable insights into international ICU nurses’ perceptions of end-of-life care in Saudi Arabia, but some limitations should be considered.

Focusing solely on international nurses’ experiences might not fully represent the practices and perspectives of Saudi Arabian nurses, potentially limiting the generalizability of findings. Additionally, conducting the study within a single hospital or region could restrict the transferability of findings to other parts of Saudi Arabia.

The study would have benefited from including the perspectives of patients, families, and Saudi Arabian healthcare providers. Understanding their experiences and beliefs would provide a more comprehensive understanding of end-of-life care practices within this specific cultural context.

The small sample size (*n* = 8) and homogeneity of participants limit the generalizability of findings. A larger, more diverse sample would increase confidence in applying results to a broader population and different contexts. This limitation may restrict the transferability of findings to other cultural contexts or healthcare systems.

## Electronic supplementary material

Below is the link to the electronic supplementary material.

Supplementary Material 1

## Data Availability

The research data includes sensitive or confidential information are available from the corresponding author on reasonable request.
