# Supplementary material for: Bridging cultural gaps in end-of-life care: the experiences of international charge nurses in Saudi Arabia
Source: BMC Nurs. 2024 Nov 28;23:865. doi: 10.1186/s12912-024-02514-7 (PMC11606103; doi:10.1186/s12912-024-02514-7)
Supplement: Supplementary file 1 — Supplementary Material 1 [file 12912_2024_2514_MOESM1_ESM.docx]

**Interview questions/prompts**

Thank you for taking the time to share your expertise with us. We believe that your experience will benefit our project about End-of-Life Care in ICU in Saudi Arabia.

Question 1 - What is good end-of-life care?

Question 2 – What are the obstacles to good end-of-life care in the ICU?

Question 3, ask the following:

a) Can you tell me a little more about how that impacts on good end-of-life care?

b) Can you give an example?

c) What strategies do nurses use to minimise the obstacle?

(If not already identified above):

Question 4 – Does culture or religion impact on your ability to provide good end- of- life care in the ICU? If so, how?

Question 5 – Does language have an effect on providing good end-of-life care? If so, how?

Question 6 – How do you feel about the quality of end-of-life care you

provide?

Question 7 – Does providing end-of-life care in ICUs have an impact on the nursing workforce? If so, how?

Question 8 – Is there anything else you would like to add?
